# Bilateral Renal Infarction Secondary to Hypertrophic Cardiomyopathy

**DOI:** 10.7759/cureus.4046

**Published:** 2019-02-11

**Authors:** Rehan Farooqi, Umar Zahid, Amrit Paudel, Durga Sivacharan Gaddam, Gavneet S Sandhu

**Affiliations:** 1 Internal Medicine, Medstar Union Memorial Hospital, Baltimore, USA; 2 Internal Medicine, Johns Hopkins Bayview Medical Center, Baltimore, USA; 3 Internal Medicine, Union Memorial Hospital, Baltimore, USA; 4 Diagnostic Radiology, University of Maryland Medical Center, Baltimore, USA

**Keywords:** acute kidney injury, bilateral renal infarction, cardiogenic etiology, bilateral renal infarction, hypertrophic obstructive cardiomyopathy, renal blood flow

## Abstract

Bilateral renal infarction is a rare phenomenon which can be difficult to diagnose because the symptoms may often mimic renal calculi, infection, muscle inflammation, genital diseases, myocardial infarction, or ischemia. We present the case of a 55-year-old male patient who presented with non-radiating, left-sided flank pain associated with nausea and vomiting. A computed tomography (CT) scan of the abdomen and pelvis with contrast demonstrated bilateral renal infarction. A thorough workup was initiated, and the thrombus formation due to left atrial enlargement from hypertrophic obstructive cardiomyopathy was considered as the cause of the bilateral renal infarction in this patient. The patient's renal function improved with treatment, and she was discharged on an anticoagulant, considering her left atrial enlargement and renal infarction.

## Introduction

Acute renal infarction is a rare entity with variable and possibly misleading manifestations. Misdiagnosis may lead to a delay in treatment resulting in permanent renal impairment or increased risk of embolism in other organs [1]. In previous studies, the incidence of renal infarction has been estimated to be 0.004% to 0.007% based on admissions to the emergency department, while the estimated prevalence in autopsy studies was 14 per 1,000 [1-3]. The infarction can result from the blockage of arterial or venous drainage, with compromised arterial supply being far more common than venous abnormalities. The incidence is higher in patients with atherosclerosis, kidney damage at baseline (nephritic syndrome, glomerulonephritis), fibromuscular disease, aneurysms, and dissections of the renal artery. Bilateral infarction was reported to be found in dissecting aneurysms of the aorta, septic emboli from endocarditis, lupus, vasculitis, sickle cell disease, or with fibromuscular dysplasia of the renal arteries [4-5]. Renal infarction in patients with a patent foramen ovale secondary to paradoxical emboli has also been reported in the literature. Herein, we report the case of a 55-year-old male diagnosed with a bilateral renal infarction caused by left atrial enlargement from hypertrophic obstructive cardiomyopathy (HOCM). To our knowledge, this is the first reported case of a bilateral renal infarction in a patient with hypertrophic obstructive cardiomyopathy.

## Case presentation

**Figure 1:**
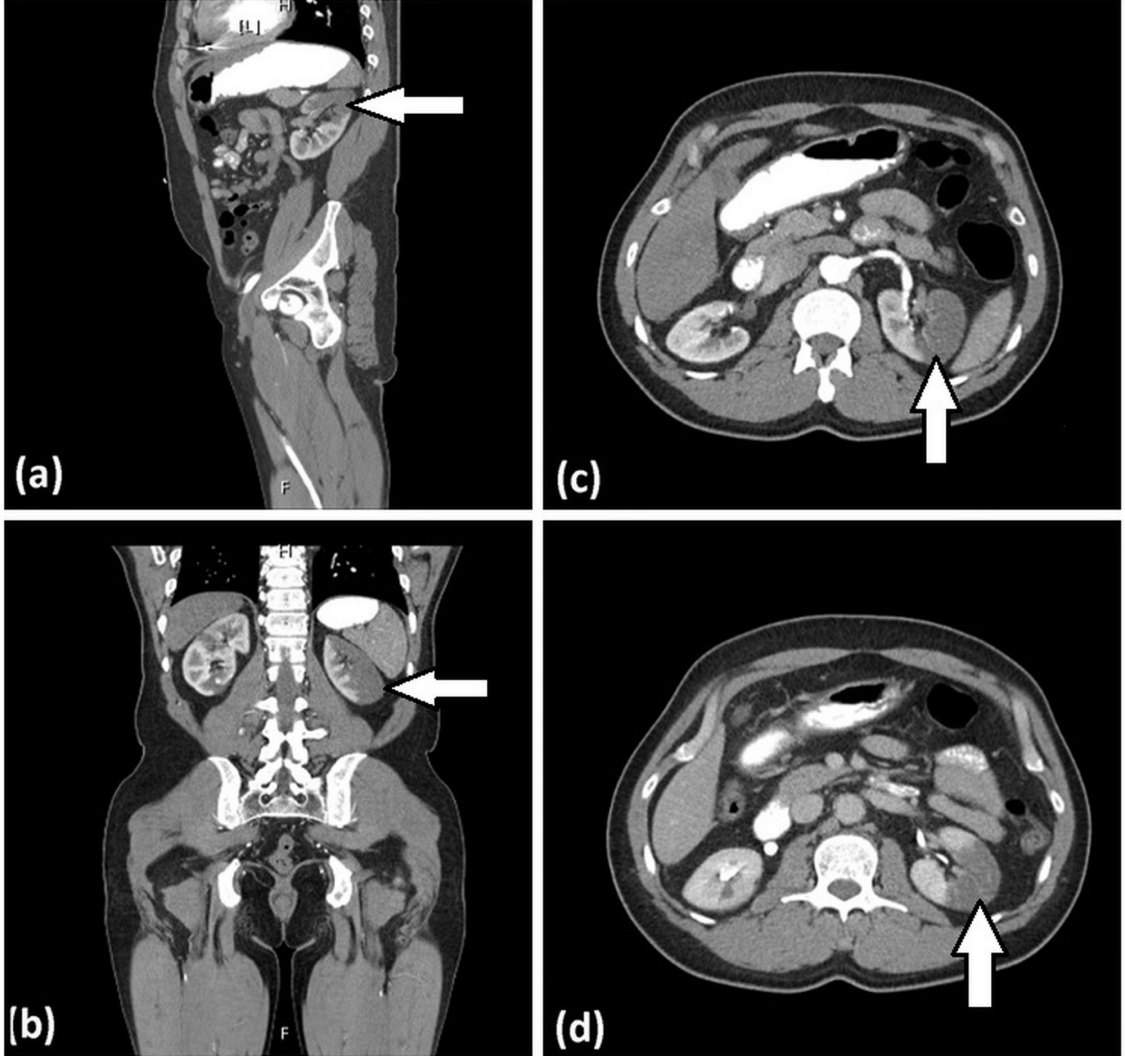
Computed tomography (CT) of the abdomen and pelvis with contrast Figure 1 shows a bilateral renal infarction. The arrows depict filling defects in the (a) sagittal, (b) coronal, and (c, d) axial images of the CT scan of the abdomen and pelvis with contrast.

**Figure 2:**
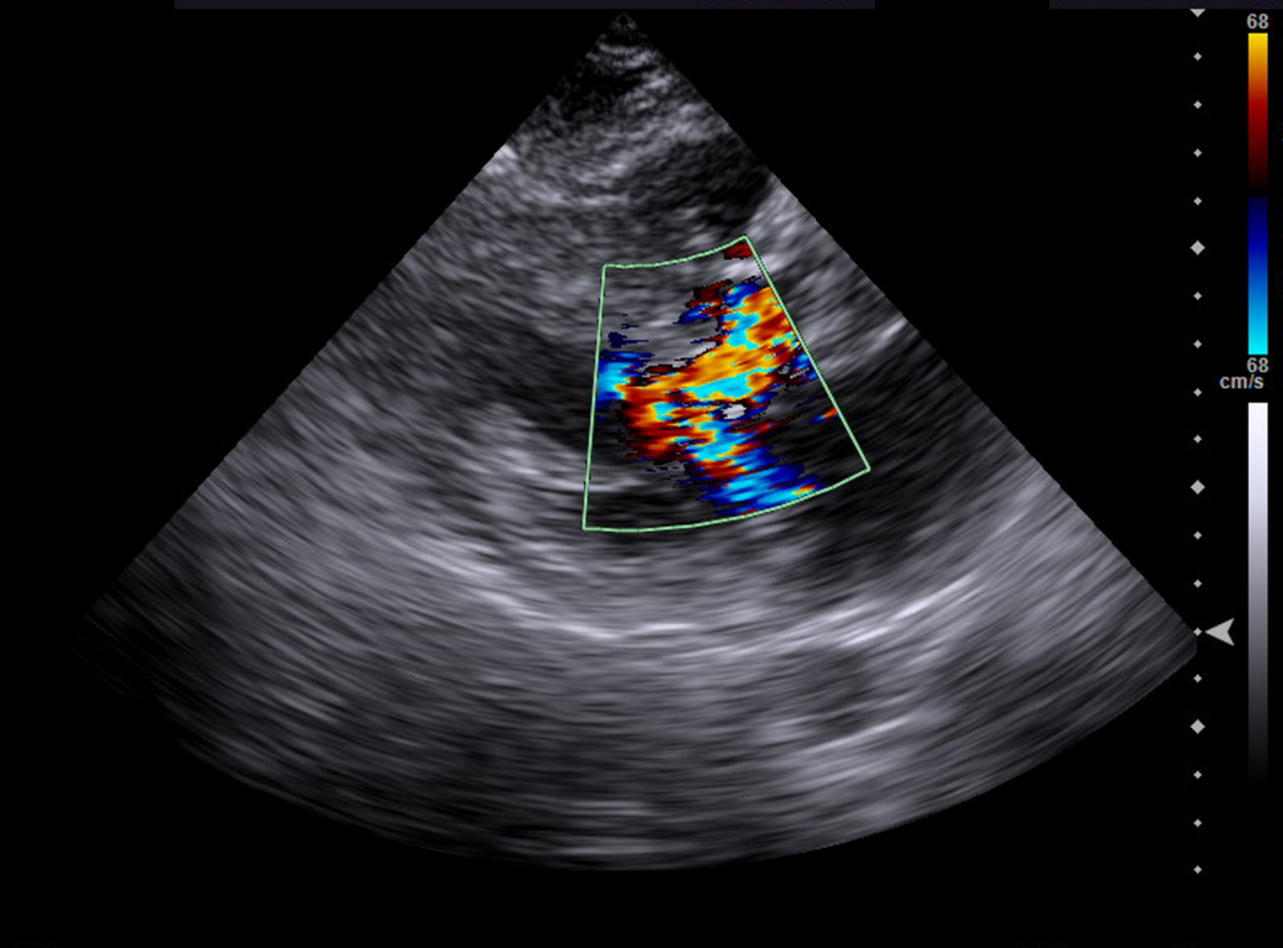
Color Doppler echocardiography Color Doppler echocardiography (systolic frame) shows marked turbulence in the left ventricular outflow tract (LVOT) and a posteriorly directed jet of mitral regurgitation. LVOT during systole is due to systolic anterior motion (SAM) of the mitral valve, resulting in a Venturi effect.

A 55-year-old male with a history of hypertension and HOCM presented to the emergency department with the acute onset of sharp, non-radiating, left-sided flank pain associated with nausea and vomiting. On admission, his vital signs were unremarkable. Physical exam was significant for a Grade III/VI systolic murmur, loudest at the apex, with no radiation. Marked tenderness on superficial palpation of the left inferior costal margin was present. There was no rebound tenderness, no costovertebral angle tenderness, and no abdominal or flank erythema. Lab workup demonstrated leukocytosis at 13,000 and acute kidney injury (creatinine: 1.3 mg/dl from a baseline of 0.7 mg/dl). Urinalysis was positive for hematuria, whereas urine toxicology was negative for any illicit substances. Computed tomography (CT) scan of the abdomen and pelvis without contrast showed no evidence of nephrolithiasis. CT scan of the abdomen and pelvis with contrast demonstrated bilateral segmental hypoperfusion indicative of a bilateral renal infarction, the left greater than the right, with no evidence of hydronephrosis (Figure 1). An electrocardiogram (EKG) upon admission showed a normal sinus rhythm with no evidence of infarction, ischemia, or atrial fibrillation. The patient was started on a heparin drip soon after the infarction was noted. Further workup ruled out infection, a hypercoagulable state (anti-cardiolipin antibody, perinuclear antineutrophil cytoplasmic antibodies (P-ANCA), cytoplasmic antineutrophil cytoplasmic antibodies (C-ANCA), protein C, protein S, antithrombin antibody, and Factor V Leiden), autoimmune etiology, sickle cell disease, patent foramen ovale, and arrhythmias. A transthoracic echocardiogram (TTE) showed hyperdynamic left ventricle systolic function, a moderately dilated left atrium at 54 mm, and mild thickening of the anterior and posterior mitral valve leaflets. Later, transesophageal echocardiography (TEE) was performed which showed a peak subvalvular gradient around 20 mmHg with no obvious masses or vegetation. A small rupture in the subvalvular chord and a left ventricular outflow tract (LVOT) obstruction was also observed (Figure 2). Different blood cultures were obtained throughout the hospital stay and no microbial organism was isolated, including bacteria, fungus, or acid-fast bacilli. Serologic antibody titers for Bartonella, Rickettsia, and M. pneumoniae were also negative. No obvious source of embolic origin was identified on echocardiogram and imaging of the renal arteries. The patient was placed on telemetry throughout his hospital course, and there was no evidence of any underlying arrhythmia, such as atrial fibrillation. However, it was presumed that the left atrial enlargement might be a predisposing factor to thromboembolic renal infarction via the same mechanism by which it predisposes to stroke [6]. The patient's renal function improved with treatment, and he was discharged on an anticoagulant, considering his left atrial enlargement and renal infarction.

## Discussion

Herein, we have presented a case of bilateral renal infarction and HOCM. The diagnosis of renal infarction may be difficult or delayed because of the rarity of the disorder and the non-specific signs and symptoms with which it presents. Symptoms can be misleading and mimic other pathology, including renal colic, mesenteric ischemia, spinal disorders, genitourinary disorders, and muscular strain. Based on the etiology, four main types of renal infarction were proposed: cardiac origin, renal artery injury, hypercoagulable state, and idiopathic [1, 7]. Renal infarction commonly results from thromboembolic events related to dysrhythmias or cardiac structural disease. The most common cause is cardiac in origin, such as secondary to atrial fibrillation. Vegetations in infective endocarditis and cardiac tumors are other possible sources of emboli.

Patients with renal infarction generally complain of flank or back pain which may be accompanied by nausea/vomiting or hematuria, along with fever. Fevers have been reported in about half of the reported cases of renal infarction [8]. Our patient’s hospital course was complicated by an acute kidney injury, fevers up to 39 degrees C, and worsening leukocytosis, all of which were attributed to renal infarction and inflammatory state. These laboratory and objective parameters improved throughout his hospital stay, and their improvement corresponded with improvement in the patient’s pain.

Vascular and hypercoagulable causes for the bilateral renal infarction were ruled out after an extensive non-revealing workup. In our patient, no obvious source of embolic origin was identified on echocardiogram nor renal arterial imaging. He had no history of atrial dysrhythmia or any events on cardiac telemetry. A TEE did reveal a ruptured subvalvular chord, which might have caused an injured endothelial surface serving as a nidus for a thrombus to form and embolize bilaterally. Another plausible cause could be attributed to the severely dilated left atrium to 54 mm from the HOCM. It is known that patients with enlargement of the left atrium are prone to develop various complications, including thrombus formation and thromboembolic events. This is because an enlarged left atrium is associated with the stasis of blood, promoting thrombus formation [1]. The risk of thromboembolism increases with increasing left atrial size regardless of the degree of anticoagulation. A review of patients from the Framingham Heart Study revealed that left atrial enlargement was a significant predictor of stroke in men [6]; thus, it is possible that left atrial enlargement is a predisposing factor to thromboembolic renal infarction via the same mechanisms by which it predisposes to stroke.

To our knowledge, there has not been a case of bilateral renal infarction in a patient with underlying HOCM reported in the literature. It appears that left atrial enlargement in a patient with HOCM increases the risk of peripheral embolism. The risk is heightened in the presence of a known bilateral renal infarction. Thus, we believed it was prudent to anticoagulate our patient. He was discharged on Rivaroxaban to reduce the likelihood of future embolic sequelae and was prescribed to take it indefinitely, given the high likelihood of a primary thromboembolic event being the cause of his renal infarction.

## Conclusions

Renal infarction is a rare entity and has various presenting symptoms which can make the diagnosis difficult. It is important to obtain a detailed history, perform a thorough physical exam, and obtain diagnostic imaging to establish the diagnosis early as any delay in treatment can result in irreversible renal impairment. To our knowledge, no cases of bilateral renal infarction secondary to thromboembolic disorders in the absence of cardiac dysrhythmia have been reported in the literature. Given the findings of the left atrial enlargement, HOCM, and bilateral renal infarction, we believe it was essential for our patient to be discharged on a lifelong anticoagulant to decrease the likelihood of future embolic sequelae.
